# Arsenic as an Endocrine Disruptor: Arsenic Disrupts Retinoic Acid Receptor–and Thyroid Hormone Receptor–Mediated Gene Regulation and Thyroid Hormone–Mediated Amphibian Tail Metamorphosis

**DOI:** 10.1289/ehp.10131

**Published:** 2007-10-26

**Authors:** Jennifer C. Davey, Athena P. Nomikos, Manida Wungjiranirun, Jenna R. Sherman, Liam Ingram, Cavus Batki, Jean P. Lariviere, Joshua W. Hamilton

**Affiliations:** Department of Pharmacology & Toxicology, and Center for Environmental Health Sciences, Dartmouth Medical School, Hanover, New Hampshire, USA

**Keywords:** arsenic (As), *CYP26A*, deiodinase (*DIO1*), endocrine, retinoic acid (RA), steroid, thyroid (TH)

## Abstract

**Background:**

Chronic exposure to excess arsenic in drinking water has been strongly associated with increased risks of multiple cancers, diabetes, heart disease, and reproductive and developmental problems in humans. We previously demonstrated that As, a potent endocrine disruptor at low, environmentally relevant levels, alters steroid signaling at the level of receptor-mediated gene regulation for all five steroid receptors.

**Objectives:**

The goal of this study was to determine whether As can also disrupt gene regulation via the retinoic acid (RA) receptor (RAR) and/or the thyroid hormone (TH) receptor (TR) and whether these effects are similar to previously observed effects on steroid regulation.

**Methods and results:**

Human embryonic NT2 or rat pituitary GH3 cells were treated with 0.01–5 μM sodium arsenite for 24 hr, with or without RA or TH, respectively, to examine effects of As on receptor-mediated gene transcription. At low, noncytotoxic doses, As significantly altered RAR-dependent gene transcription of a transfected RAR response element–luciferase construct and the native RA-inducible cytochrome P450 *CYP26A* gene in NT2 cells. Likewise, low-dose As significantly altered expression of a transfected TR response element–luciferase construct and the endogenous TR-regulated type I deiodinase (*DIO1*) gene in a similar manner in GH3 cells. An amphibian *ex vivo* tail metamorphosis assay was used to examine whether endocrine disruption by low-dose As could have specific pathophysiologic consequences, because tail metamorphosis is tightly controlled by TH through TR. TH-dependent tail shrinkage was inhibited in a dose-dependent manner by 0.1– 4.0 μM As.

**Conclusions:**

As had similar effects on RAR- and TR-mediated gene regulation as those previously observed for the steroid receptors, suggesting a common mechanism or action. Arsenic also profoundly affected a TR-dependent developmental process in a model animal system at very low concentrations. Because RAR and TH are critical for both normal human development and adult function and their dysregulation is associated with many disease processes, disruption of these hormone receptor–dependent processes by As is also potentially relevant to human developmental problems and disease risk.

Exposure to excess arsenic, principally from contaminated drinking water, is considered one of the top environmental health threats both in the United States and worldwide [Abernathy et al. 2003; Mukherjee et al. 2006; National Research Council (NRC) 1999, 2001; Smith et al. 2002; Watanabe et al. 2003]. The majority of this exposure is from natural geological sources of As that contaminate groundwater. Epidemiologic studies have linked chronic exposure to drinking-water As with increased risks of various cancers, including those of the lung, bladder, skin, and liver, as well as numerous other noncancer illnesses including vascular and cardiovascular disease, diabetes, developmental and reproductive problems, and neurologic and cognitive problems (Abernathy et al. 2003; NRC 1999, 2001; Smith et al. 2002; Tapio and Grosche 2006; Wasserman et al. 2004; Watanabe et al. 2003). Public water supplies in the United States and Europe currently have a regulatory limit of 10 ppb As (0.13 μM). However, a large segment of the population in the United States and worldwide obtains its drinking water from private unregulated wells. Also, many other areas of the world have higher regulatory limits or water supplies that are unregulated. Thus, As contamination continues to be a serious, ongoing public environmental health problem affecting hundreds of millions of people.

There are a number of proposed mechanisms for the ability of As to influence so many diverse disease processes. These include alterations in cell signaling, cell cycle control, oxidative stress, DNA repair, and others (Abernathy et al. 1999; Aposhian and Aposhian 2006; Kitchin 2001; Rossman 2003). Moreover, it is becoming clear that there are important dose-, time-, and tissue-specific differences in effects of As, as well as important gene–environment and co-exposure interactions that complicate how As alters disease risk under any particular exposure circumstance (Andrew et al. 2003, 2006; Bodwell et al. 2004, 2006; Karagas et al. 2004; Waalkes et al. 2003). We previously reported that As is a potent endocrine disruptor, altering steroid hormone receptor (SR)-mediated gene regulation at very low, environmentally relevant concentrations in cell culture and in whole-animal models (Bodwell et al. 2004, 2006; Davey et al. 2007; Kaltreider et al. 2001). We have demonstrated that all five steroid receptors (SRs) [i.e., the receptors for glucocorticoid (GR), androgen (AR), progesterone (PR), mineralocorticoid (MR), and estrogen (ER) hormones] are affected in a similar manner, suggesting a broad effect on these pathways and also suggesting a common mechanism for these effects (Bodwell et al. 2004, 2006; Davey et al. 2007; Kaltreider et al. 2001). Given this strong disruption on this entire class of nuclear hormone receptors, we were interested in whether these effects extend to other members of the larger nuclear hormone receptor superfamily.

In this study we examined the effects of As on gene regulation by two related class II receptors, the retinoic acid (RA) receptor (RAR) and the thyroid hormone (TH) receptor (TR). Both receptors normally partner with the retinoid X receptor (RXR) to form heterodimers that act as transcription factors, binding to the RA-response element (RARE) of RA-inducible genes or the TH-responsive element (TRE) of TH-inducible genes, respectively (Mark et al. 2006; Tsai and O’Malley 1994; Wong and Shi 1995; Zhang and Lazar 2000). We report here that As has significant effects on both RAR- and TR-mediated gene regulation at very low doses (0.01–2 μM), and that these effects are strikingly similar to those previously observed with the SRs, suggesting a common mechanism. We also investigated whether such As-induced alterations in hormone signaling would lead to pathophysiologic consequences, using amphibian tail metamorphosis as a model system. Anuran metamorphosis is highly dependent on TH- and TR-mediated processes (Buchholz et al. 2006; Buckbinder and Brown 1993; Wong and Shi 1995; Yaoita and Brown 1990) and is known to be perturbed by agents that interfere with TH or TR signaling (Buchholz et al. 2003; Lim et al. 2002). Because TH is also important for many aspects of mammalian embryonic development (Galton 2005; Oppenheimer and Samuels 1983; Porterfield and Hendrich 1993), this is a useful model for understanding possible effects on human development. Arsenic had profound effects on TH-mediated *ex vivo* tail metamorphosis at very low, environmentally relevant concentrations, suggesting that there are likely to be other developmental and pathophysiologic effects of low dose As endocrine disruption *in vivo*.

## Methods

### NT2 cell culture and transfections

NT2 [NTERA-2; American Type Culture Collection (ATCC), Manassas, VA] human embryonic carcinoma cells were maintained in Dulbecco’s Modification of Eagle’s Medium (DMEM; Invitrogen, Carlsbad, CA) plus 10% fetal bovine serum (FBS; Atlanta Biologicals, Norcross, GA). Cells were split into 6-well plates at 1.5 × 10^5^ cells/well and grown in phenol red–free DMEM plus 10% charcoal-stripped FBS overnight for transfection experiments. The construct used was generously provided by James DiRenzo (Dartmouth Medical School). Briefly, the construct contains two canonical tandem RARE sequences (5′-AGGTCA-(N)_5_-AGGTCA-3′) upstream of a thymidine kinase promoter and the firefly luciferase coding region with a pBR322 backbone (RARE-LUC) (Kurokawa et al. 1994). Cells were transfected with 250 ng of the RARE-LUC construct using Fugene (Invitrogen) according to the manufacturer’s recommended protocol. All-trans-retinoic acid (ATRA; Sigma Chemical Co., St. Louis, MO), reconstituted in DMSO and stored at –20°C, was used as ligand at concentrations indicated. Arsenic (As, As^+3^, sodium arsenite, NaAsO_2_; Sigma Chemical Co.) was dissolved in water and kept frozen until the day of use. Treatments were performed 24 hr after transfection. ATRA and As were added simultaneously at the doses and for the durations described in “Results.”

### Clonogenic cell survival assay for NT2 cells

NT2 cells were grown in phenol red–free DMEM media plus 10% FBS and split into 6-well plates with approximately 100 cells/well and allowed to grow overnight. Cells were then exposed to 1.0–7.0 μM As plus 10 nM ATRA for 24 hr. Next, fresh media without As but including 10 nM ATRA was added, and cell growth was checked daily. When colonies of ≥ 25 cells were formed in control wells, the media was removed and the cells were fixed in 1% formaldehyde for 1 hr and then exposed to 100% methanol for 5 min. Methanol was removed and cells were stained with 0.3% Giemsa stain (in 70% methanol) for 15 min. Colonies were rinsed twice with phosphate-buffered saline (PBS) and counted. Results are given as a percent of control, and each condition was repeated in triplicate.

### GH3 cell culture and transfections

GH3 rat pituitary tumor cells (ATCC) were cultured in F-12 Nutrient Mixture (Ham) (Invitrogen) supplemented with 15% horse serum (Atlanta Biologicals) and 2.5% FBS. For triiodo-thyronine (T_3_)/As treatments, 6-well plates were seeded at 350,000–750,000 cells/well and cultured in F-12 medium for 3 days. Cells were then put into phenol red–free DMEM supplemented with 10% charcoal-stripped FBS for 18 hr prior to treatments. The TR response element–luciferase (TRE-luc) construct used for transfections was created by exchanging the glucocorticoid response element (GRE) sequence with two canonical TRE direct repeats in the pXP2-GRE–luciferase construct kindly contributed by J. Bodwell (Dartmouth Medical School). The final construct was sequenced to verify successful insertion. Transfections were performed with Lipofectamine Plus (Invitrogen) with the following optimizations. Twenty-four hours after plating, media was changed to unsupplemented Opti-MEM (Invitrogen), then transfection followed using the manufacturer’s protocol. Three hours later, Opti-MEM was replaced with phenol red–free DMEM plus 10% charcoal-stripped FBS for the duration of the experiment. Cells were treated with As as indicated. T_3_ (CAS 55-06-1; Sigma) was reconstituted to 1 mM in 45% propylene glycol (1,2-propanediol; Aldrich, St. Louis, MO), 45% water, and 10% 0.1 N sodium hydroxide and stored at 4°C covered.

### Clonogenic cell survival assay for GH3 cells

Clonogenic assays were performed by plating approximately 5,000 cells/well in a 6-well plate and allowing them to grow in F-12 Nutrient Mixture for a total of 3–4 days. After an 18-hr treatment in phenol red–free DMEM with 10% charcoal-stripped serum, cells were treated for 24 hr with various doses of As, with or without T_3_. Arsenic was removed and the cells were allowed to grow for approximately 5 days in phenol red–free DMEM plus 10% charcoal-stripped serum with or without T_3_. Cells were trypsinized, resuspended in PBS, and counted in azide-free isotonic diluent (Valtech Diagnostics, Inc., Brackenridge, PA) using a Coulter Counter Z1 (Beckman Coulter, Fullerton, CA). Cells were counted individually rather than via colony formation (as with the NT2 cells) because GH3 cells would not adhere under the conditions necessary for staining the colonies.

### ICP-MS analysis of total arsenic in cell culture media

Total arsenic levels in cell culture media were measured by the Dartmouth Superfund Trace Metal Core Facility using collision cell inductively coupled plasma mass spectroscopy (ICP-MS; Agilent octopole/reaction cell 7500c ICP-MS; Agilent, Palo Alto, CA), employing helium as the collision gas. The media was diluted and analyzed by the method of standard additions. Method detection limits were 0.005 μM, and the overall method uncertainty was 8%. All media used contained undetectable levels of As or levels well below any of the exogenously added effective doses (i.e., < 0.01 μM).

### Luciferase and protein assays

Cells were rinsed twice with cold PBS, covered with 150 μl of Promega lysis buffer, and scraped from the plate. Cell lysates were frozen at –80°C, thawed, vortexed for 30 sec, and spun at 12,000 x *g* for 5 min; supernatants were then transferred to a new tube and stored at –80°C. Each experimental condition in each experiment was done in replicates of six. Transfection efficiency was consistent, with little variability among the biological repeats in each treatment group and low standard deviations between 3 to 4 replicate experiments. We used Prism software (GraphPad Software Inc., San Diego, CA) for all statistical analyses. Luciferase assays were performed with the Promega Luciferase Assay System kit (Promega, Madison, WI) following the manufacturer’s recommended protocol. We used a luminometer (Dynatech Laboratories, Chantilly, VA) to determine the light units per sample. Protein assays were performed with the Pierce BCA Protein Assay Reagent Kit (Pierce, Rockford, IL) following manufacturer’s recommended protocol to normalize the total level of protein in samples. Results of protein assays were determined using a Thermomax microplate reader (Molecular Devices, Sunnyvale, CA).

### Semiquantitative real-time polymerase chain reaction (PCR) for CYP26A or DIO1

We isolated total RNA from the cells after indicated treatment times using the Qiashredder and RNeasy Mini Kits (Qiagen, Valencia, CA) or Trizol (Invitrogen) according to the manufacturer’s recommended protocols. Contaminating genomic DNA was removed using DNA-free kits (Ambion, Austin, TX), and total RNA was quantified with a NanoDrop ND-1000 Spectrophotometer (NanoDrop Technologies, Rockland, DE). RNA (1–2 μg) was reverse-transcribed (gene-specific primer) with Omniscript Reverse Transcriptase (Qiagen), and primers were synthesized to specifically amplify either the rat type I deiodinase (*DIO1*) gene from the GH3 cells (GenBank accession no.BC083557; National Library of Medicine 2007) or the human cytochrome P450 26A (*CYP26A*) gene from the NT2 cells (GenBank accession no. NM000783) [see Supplemental Material (http://www.ehponline.org/members/2007/10131/suppl.pdf) for details]. We performed semiquantitative real-time PCR (RT-PCR) to assess relative quantities of each transcript with the 7500 Real-Time PCR System (Applied Biosystems, Foster City, CA). Negative controls, including samples that would either detect contaminating genomic DNA in the RNA samples or contamination of PCR reagents, were always included. On each plate we included an internal standard curve for each transcript, which consisted of a serial dilution of cDNA known to contain the transcript in question. The curve generated from the plate was then used to quantify the level of transcript for all the experimental samples on the same plate. RT-PCR for 18S levels was performed to normalize total RNA levels. Results were unaltered by 18S normalization.

### *Xenopus* tadpole culture

We purchased *Xenopus laevis* tadpoles from Nasco (Fort Atkinson, WI). Animals were treated humanely and with regard to alleviation of suffering; all studies were performed in compliance with institutional animal care and use guidelines approved by Dartmouth Medical School (Protocol 04-03-11). Animals were acclimated to COMBO culture media (8.7 mg/L K_2_HPO_4,_ 36.9 mg/L MgSO_4_, 62.8 mg/L NaNO_3_, 36.7 mg/L CaCl_2_, 28.42 mg/L Na_2_SiO_3_, 24 mg/L H_3_BO_4_, 12.6 mg/L NaHCO_3_ plus 2.18 μg/L H_2_SeO_3_, 16 μg/L NaBr, 155 μg/L LiCl, 70 μg/L RbCl, 3.3 μg/L KI, 150 μg/L SrCl_2_· 6H_2_O, pH 7.7) and carefully staged when they arrived. Animals were kept in COMBO media in 5-gal Nalgene buckets (Nalge Nunc International, Rochester, NY) at room temperature until they reached stage 58 of development. Stage 58 was determined using staging criteria determined by Nieuwkoop and Faber (1994). At stage 58, *Xenopus* tadpoles have functional TR in tail tissue, and the tail will metamorphose upon exposure to T_3_ whether or not it is attached to the animal (Shi et al. 2002; Tata 1966).

### *Ex-vivo* tail culture

Tails were excised from stage 58 tadpoles, dipped in 70% ethanol, and immediately placed in a 6-well plate with 3.0 mL phenol red–free minimal essential media (MEM; Invitrogen) per well. Arsenic was added to the media immediately (doses indicated in the “Results”), and T_3_ was added to the media 18 hr later. Trial experiments in which arsenic and T_3_ were added simultaneously resulted in similar shrinkage patterns. Tail cultures were kept in the dark at approximately 20–23°C. Medium was changed daily, and photographs were taken with a digital Nikon Coolpix camera (Nikon Inc., Melville, NY). Experiments lasted approximately 5 days. Fins of tails treated with T_3_ only generally resorbed significantly or completely in 4 days. Digital photographs were analyzed with NIH Image software (National Institutes of Health, Bethesda, MD) to assess the area of tail fin each day. The area of the dorsal tail fin on day 4 was divided by the area of the dorsal tail fin on day 1 to determine the percent of resorption. Each treatment group in each experiment consisted of 6 tails, and the experiment was repeated four to eight times. Occasionally tails would not resorb at all when given T_3_ alone, indicating that they did not have sufficient TR to metamorphose, most likely as a result of incorrect staging. In these cases, all tails in the experiment that did not resorb at all when given T_3_ were removed from analysis regardless of treatment; 3%–10% of the 42 tails in an experiment were removed and they were evenly distributed between the groups. Statistical significance was determined with Prism software using an unpaired *t*-test and a *p*-value < 0.01 for the tail fin area, except when determining the optimal T_3_ dose where the value shown is mean ± SE.

## Results

### Effects of As on RAR-mediated transcription

**Figure 1:**
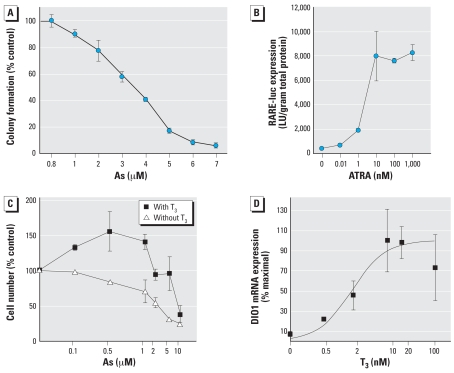
Dose–response curves for As, ATRA, and TH in NT2 and GH3 cells calculated using average values for each dose. Data points represent the mean ± SE of data from three separate experiments. (*A*) Cytotoxicity of As in NT2 cells exposed to As for 24 hr and assessed by colony-forming assay. Data are expressed as colony formation as a percent of the control. (*B*) Induction of RARE-luc expression in NT2 cells by ATRA. RARE-luc expression is expressed as mean ± SE luciferase (LU) per gram protein. The median effective concentration (EC_50_) for ATRA induction is approximately 7 nM. (*C*) Cytotoxicity of As in GH3 cells assessed essentially as described for (*A*), except that cells were cultured in media plus stripped serum with or without 10 nM T_3_. (*D*) Induction of *DIO1* expression by T_3_ in GH3 cells assessed essentially as described in (*C*), except that DIO1 mRNA was assessed by RT-PCR. Data are expressed as a percent of the maximum value. See “Methods” for details.

**Figure 2:**
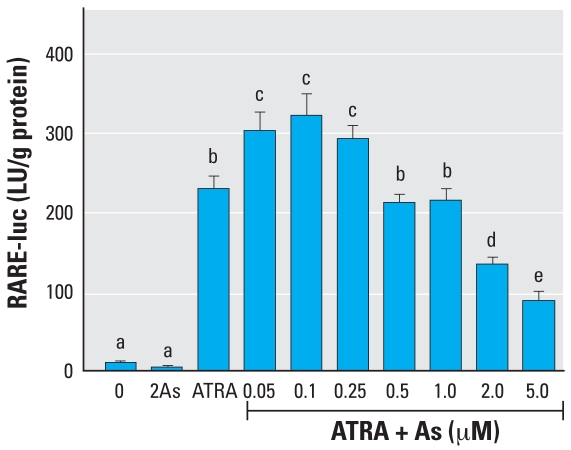
Effects of As on ATRA induction of RARE-luc expression in NT2 cells. Cells were transfected with the RARE-luc construct 24 hr before treatment with 10 nM ATRA with or without simultaneous treatment with As for 24 hr. See “Methods” for details. Data are expressed as mean ± SE of the values from replicates of experiments. Bars that do not have the same letter are significantly different from each other at *p* < 0.003 using an unpaired *t*-test.

**Figure 3:**
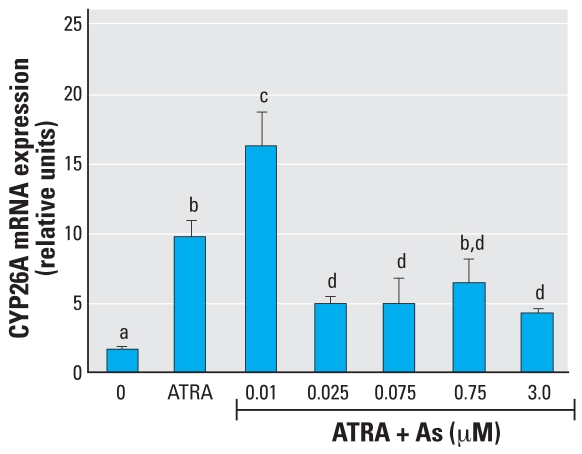
Effects of As on ATRA induction of CYP26A mRNA expression in NT2 cells. Cells were treated with 10 nM ATRA with or without simultaneous addition of As for 24 hr; mRNA expression was measured by RT-PCR. See “Methods” for details. Data are expressed as mean ± SE of the values from replicates of experiments. Bars that do not have the same letter are significantly different from each other at *p* < 0.003 using an unpaired *t*-test.

Ligand-activated RAR forms heterodimers with RXR and binds RAREs in RA-responsive gene promoters, which induces transcription (Mark et al. 2006). Initial dose–response experiments (Figure 1B) demonstrated that 10 nM ATRA was an adequate and physiologically relevant dose of ligand that induces a significant increase in expression of the RARE-luc construct when transfected into these cells. Cytotoxicity, measured by colony-forming assays, demonstrated an LC_50_ (concentration lethal to 50%) of 3 μM for NT2 cells (Figure 1A). Very low concentrations of As (0.05–0.25 μM) enhanced RA-inducible RARE-luc expression compared with RA alone (Figure 2). Conversely, a higher but still non-cytotoxic concentration of 2.0 μM As strongly repressed RAR-mediated induction of the construct (as did the higher but slightly cytotoxic dose of 5 μM). The *CYP26A* gene is endogenously expressed in NT2 cells, and a RARE in its promoter facilitates its induction by ATRA (Loudig et al. 2000). The transcript level of the *CYP26A* gene was measured by RT-PCR in NT2 cells after exposure to 10 nM ATRA plus As (Figure 3). Similar to the RARE-luc construct, hormone induction of *CYP26A* mRNA expression was enhanced by 10 nM ATRA plus As at a very low dose (0.01 μM), and its induction was repressed in a dose-dependent manner by As doses of ≥ 0.025 μM.

### TH-mediated transcription

**Figure 4:**
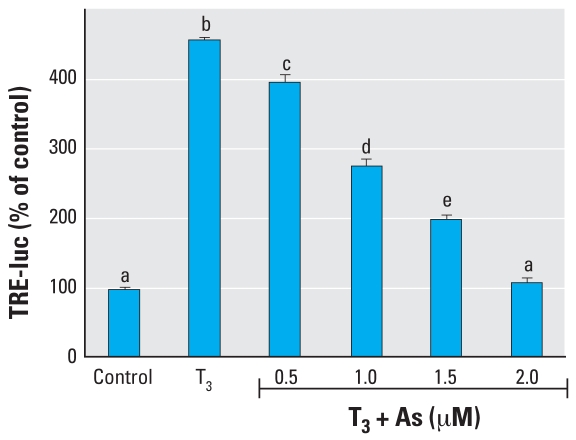
Effects of As on T_3_ induction of TRE-luc expression in GH3 cells. Cells were transfected with the TRE-luc construct 24 hr before treatment with 2 nM T_3_ with or without simultaneous treatment with As for 24 hr. See “Methods” for details. Data are expressed as mean ± SE of the values from replicates of experiments. Bars that do not have the same letter are significantly different from each other at *p* < 0.003 using an unpaired *t*-test.

**Figure 5:**
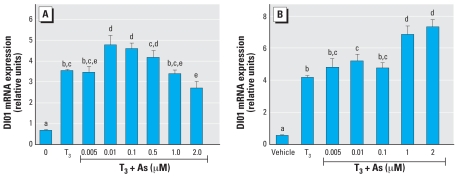
Effects of As on T_3_ induction of DIO1 mRNA expression in GH3 cells. Cells were treated with 2 nM T_3_ with or without As, and DIO1 mRNA expression was measured 6 hr (*A*) or 24 hr (*B*) after treatment. See “Methods” for details. Data are expressed as mean ± SE of the values from replicates of experiments. Bars that do not have the same letter are significantly different from each other at *p* < 0.01 using pairwise Student’s *t*-test analysis.

GH3 cells are a rat pituitary tumor cell line that expresses functional TRs and expresses genes directly induced by TH. We performed a series of initial dose–response experiments to determine an effective and physiologic dose of TH (administered as T_3_) and found that 1–2 nM T_3_ was sufficient to induce transcription (Figure 1D) of the *DIO1* gene approximately 4-fold. This was also sufficient to induce the TRE-luc construct 4-fold. GH3 cells, like the NT2 cells, were moderately sensitive to the toxic effects of As, with an LC_50_ of 5–10 μM, as determined by a clonogenic cell survival assay (Figure 1C). Interestingly, the clonogenic assay results indicated an increase in proliferation rather than cytotoxicity when 0.1–1 μM As was combined with T_3_ (the proliferative effect was removed when T_3_ was removed). The LC_50_ was decreased to 0.9–2.0 μM if the cells were grown in stripped serum without T_3_. The TRE-luc construct was repressed in a dose-dependent manner by 0.5–2.0 μM As (Figure 4). We then examined the effects of As on TH induction of the endogenously expressed *DIO1* (Jakobs et al. 1997) by measuring mRNA levels by RT-PCR 6 or 24 hr after treatment. At 6 hr, concentrations of 0.01–2.0 μM As produced a biphasic dose response similar to the NT2 results with RA, with a significant super-induction at lower As concentrations (0.01–0.1 μM) and repression at 2 μM (Figure 5A). The response at 24 hr differed from the RAR-mediated genes in that any repressive effects were gone, and 1.0 and 2.0 μM As enhanced hormone-stimulated expression. Arsenic doses > 2 μM produced significant cytotoxicity, so we could not determine whether As suppresses TH induction at 24 hr independent of general toxic mechanisms that might compromise mRNA expression.

### TH-directed amphibian tail metamorphosis

**Figure 6:**
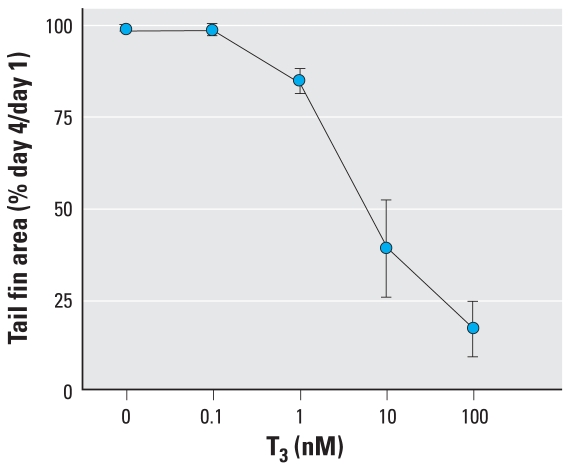
Effects of T_3_ on tail fin shrinkage of *Xenopus* tadpole tails cultured *ex vivo* as described in “Methods.” Data are expressed as mean ± SE of values from six tails per treatment in three separate experiments.

**Figure 7:**
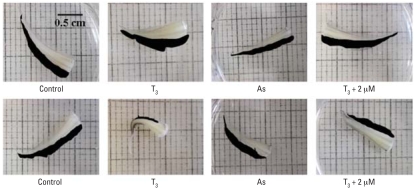
Effects of As on T_3_-mediated tail shrinkage in *Xenopus* tadpole tails cultured *ex vivo* shown by representive samples from tail resorption experiments. See “Methods” for details. Morphometric software was used to trace the tail fin area (shown in black), which was used to calculate the differences in area for each tail between day 1 and day 4. Results from these experiments are shown in Figures 6 and 8.

**Figure 8:**
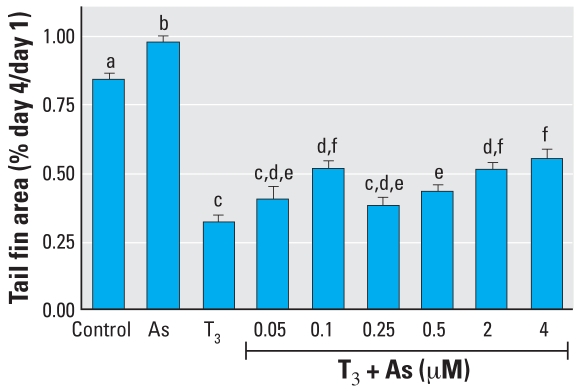
Effects of As on T_3_-mediated tail shrinkage in *Xenopus* tadpole tails cultured *ex vivo*. See “Methods” for details. Data are expressed as mean + SE of values from 5–6 individual tails per experiment and 4–8 individual experiments per treatment. Bars that do not have the same letter are significantly different from each other at *p* < 0.01 using pairwise Student’s *t*-test analysis.

Based on the effects of As on TR-mediated gene regulation described above, we were interested in whether there were specific patho-physiologic consequences of this endocrine disruption. We assessed T_3_-dependent tail shrinkage by measuring the area of the tail fin over a 4-day period, which is generally the time it takes for the fin to completely resorb in our assay. Fin resorption is the first step in visible tail metamorphosis, is amenable to quantitation by morphometric analysis, and is representative of the overall tail shrinkage process. A 10-nM dose of T_3_ in the tail culture media was consistently sufficient to induce tail fin resorption (Figure 6); thus, we used this dose throughout the subsequent experiments. As shown in Figure 7, treatment of tails *ex vivo* with T_3_ led to shrinkage of the tail fins that was readily apparent. Control tail fins not exposed to T_3_ or As normally shrank 10–20% during an experiment (Figures 7 and 8). Tails exposed to As alone shrank slightly less than controls (Figure 8). In the As plus T_3_ groups, the lowest As concentration used (0.05 μM) had little effect compared with T_3_ alone, and the tail fins resorbed approximately 58% in both groups (Figures 7 and 8). However, concentrations of As above this (0.5–4 μM) decreased T_3_-dependent tail fin resorption in a dose-dependent manner (Figure 8). A concentration of 0.25 μM As slowed resorption by 4% compared with T_3_ alone, whereas 4 μM As decreased resorption by 20%. Interestingly, the intermediate concentration of 0.1 μM As did not follow the dose-dependent pattern of the higher As doses; this finding is discussed below. In summary, As significantly altered the T_3_-and TR-dependent metamorphosis of *Xenopus* tails *ex vivo*, indicating that As disrupts T_3_ hormone signaling through its receptor, TR/RXR, and has the potential to affect hormone-regulated embryonic development at low physiologically and environmentally relevant concentrations *in vivo*.

## Discussion

Based on our previous studies with SRs, we were interested in whether As could also disrupt other, less similar nuclear hormone receptors. Because we were interested in examining the effects of As on hormone regulation of both transiently transfected gene constructs as well as native hormone-responsive genes, we chose two different cell culture models, each optimal for the receptor system under study. Our previous results (Davey et al. 2007), as well as those of others (Kaltreider et al. 2001), strongly indicate that working in the appropriate cell line is critical to mimicking the effects of As on gene expression observed *in vivo.* Mann et al. (2005) reported that As had no effect on ER-mediated gene expression in heterologous systems such as COS1 cells, which do not normally express ER but require co-transfection of an ER-expressing plasmid. Lack of response in such systems, even when the receptor is reexpressed, may indicate that other critical aspects of the receptor machinery (e.g., key coregulators that are critical for the As response) may be missing. Also, the sensitivity of cultured cells to the cytotoxic effects of As is highly cell line specific; thus, it is important to know the dose response to As for a given cell line so the appropriate dose range can be selected and the toxic-equivalent doses between lines can be compared. In the present study we found that NT2 cells are more sensitive to the toxic effects of As than other cell lines we have used. Also, in order to examine the pathophysiologic consequences of endocrine disruption of one of these type II receptors by As, we chose another well-characterized system, amphibian tail metamorphosis, which is known to be highly TR-dependent (Brown et al. 1996; Das et al. 2006; Dodd and Dodd 1976; Lim et al. 2002; Veldhoen and Helbing 2001).

We observed significant effects of As at very low, noncytotoxic, environmentally relevant concentrations on both RAR- and TR-mediated gene expression in this study. These As effects were very similar to those previously observed for GR and the other SRs, suggesting a common mechanism of action. As with SRs, the effects of As exhibited a complex dose–response pattern that was biphasic, with As significantly enhancing hormone-mediated gene expression at very low doses while strongly suppressing gene expression at higher, but still moderate, doses of As. Similar effects were observed for GR, MR, PR, AR, and ER, although the precise concentrations varied as a function of relative As toxicity in each cell system (Bodwell et al. 2004, 2006; Davey et al. 2007). Our previous studies with SRs strongly suggest that the receptors themselves are not the actual targets for these As effects. First, the SRs share little absolute homology, yet they respond almost identically to As (Bodwell et al. 2006; Davey et al. 2007). Although GR, MR, AR, and PR share extensive similarity, ER is much more distally related and shares little absolute homology, but ER responds similarly to As (Davey et al. 2007). Second, mutational studies with GR indicated that neither the N-terminal regulatory or C-terminal ligand-binding domains were necessary to elicit the effects of As (Bodwell et al. 2004, 2006). Likewise, extensive mutational analysis of the remaining central DNA-binding domain of GR failed to demonstrate any critical region that could serve as the likely target for As, nor is there sufficient homology among the SRs to explain a common As effect. The present results show similar effects of As on TR and RAR in spite of their even greater divergence from the SRs; this further supports the hypothesis that it is some common aspect of their regulation that is the actual target.

The mechanism of gene activation for TR and RAR differs from that of the SRs. Unlike the SRs, which form homodimers and bind to palindromic hormone-responsive elements (HREs) in promoters, TR and RAR each normally form obligate heterodimers with the RXR to form a functional transcription factor. The TR-RXRs and RAR-RXRs are also normally bound to their cognate response elements (TRE and RARE, respectively) in hormone-responsive promoters in the absence of ligand, unlike the SRs, which normally reside as quiescent monomers in the cytoplasm (GR, MR, PR, AR) or nucleus (ER) and then migrate to their respective HREs following hormone activation and dimerization. Co-repressors normally prevent transactivation of TR or RAR until ligand binding occurs (Bastien and Rochette-Egly 2004; Hermanson et al. 2002; McKenna and O’Malley 2002; Shi et al. 2002). Similar to what was observed for SRs, As produced a biphasic response to gene induction with RAR and TR. The suppressive effect of intermediate doses of As is seen universally and strongly with all of these nuclear hormone receptors under virtually all experimental conditions, and it appears to be most closely tied to transcription. In contrast, the low-dose As enhancement of hormone induction is more variable among receptors, is more susceptible to experimental manipulation, and appears to be most closely associated with earlier steps of receptor activation, based on detailed studies with GR (Bodwell et al. 2004, 2006). For example, certain GR mutants lack the low-dose enhancement while retaining intermediate dose suppression by As. Similarly, the low-dose enhancement can be progressively dampened and eventually lost by progressively increasing the number of hormone-activated receptors in the cell, although higher dose suppression by As remains relatively constant (Bodwell et al. 2004). We propose that the mechanism by which As enhances hormone-induced gene activation at very low doses is distinct, and can be separated experimentally, from the suppressive effects seen at the slightly higher but still noncytotoxic intermediate doses. However, the precise mechanisms by which As elicits these two effects remains to be determined.

RAR mediates RA signaling during embryonic development. Either excessive or deficient levels of RA, a derivative of vitamin A, results in teratogenic effects (Shenefelt 1972; Thompson et al. 1964; Wolbach and Howe 1978). RA signaling is also involved in tissue homeostasis, lipid metabolism, and cellular differentiation and proliferation in the adult (Shenefelt 1972). The family of retinaldehyde dehydrogenases and Cyp26(A1,B1,C1) are RA-synthesizing and RA-catabolizing enzymes, respectively. Transgenic mice without *CYP26A1* result in embryonic lethal phenotypes, thus indicating a key role of *CYP26A1* in embryogenesis (Abu-Abed et al. 2001). The present study indicates that RA-dependent CYP26A1 mRNA expression can be enhanced or repressed by As exposure, depending on the dose of As. *CYP26A1* plays a key role in inactivating RA; therefore, its dysregulation could have important pathophysiogic consequences, including developmental processes, similar to those of the *DIO1* gene (White et al. 1996, 1997). Because these effects were observed for concentrations of As that are directly relevant to environmental levels of As in drinking water of concern throughout the United States and many other parts of the world, this has potentially important implications for human reproduction and development in exposed populations.

TR is the mediator of critical TH-regulated processes in adults and during embryonic and fetal development (Gothe et al. 1999). The active form of TH, T_3_, is produced when type I or type II deiodinase removes a specific outer ring iodine of thyroxine (T_4_). T_3_ is the active TH ligand that binds TR, and therefore its production is a crucial step in TH-driven gene induction. The *DIO1* gene has a TRE in its promoter and is directly induced by T_3_ through TR. The change in expression of *DIO1* we observed at 6 or 24 hr of As exposure indicates a transient superinduction by As at very low doses and a transient repression by As at higher doses. Differential effects of acute versus chronic As exposure are of interest because TH levels—kept in equilibrium by deiodinases and T_3_ levels in the pituitary—are critical to thyroid function in the whole body. Thus, understanding how As alters T_3_ homeostasis will be an important aspect of understanding the overall effects of As on this hormone pathway, both for human development and in adult physiologic processes.

The clonogenic assays with the GH3 cells indicated enhancement of proliferation at low-dose As in the presence of T_3_. T_3_ activates growth hormone in GH3 cells, causing proliferation (Kitagawa et al. 1987; Kitamura et al. 2002), and low-dose arsenic has also been shown to cause proliferation of cells (Hwang et al. 2006; Vogt and Rossman 2001). Low-dose As appears to enhance the proliferative effect of T_3_, demonstrating another type of As-induced endocrine disruption (i.e., enhancement of proliferation that is hormone dependent).

Metamorphosis of *Xenopus laevis* has been extensively characterized (Buchholz et al. 2006; Buckbinder and Brown 1993; Gudernatch 1912; Wong and Shi 1995; Yaoita and Brown 1990). *In vivo*, the tadpole produces T_3_ in concert with induction of TR, leading to TH-dependent alterations in gene expression that regulate tail resorption (Brown et al. 1996; Das et al. 2006; Dodd and Dodd 1976). This can be mimicked by culturing appropriately staged tadpole tails *ex vivo* and exposing them to exogenous TH, leading to tail shrinkage similar to that seen *in vivo* (Das et al. 2006; Lim et al. 2002; Tata 1966; Veldhoen and Helbing 2001). Tadpole tails will respond to T_3_ in the culture media essentially as they would as part of the whole animal, by shrinking through what is thought to be a combination of apoptosis and necrosis (Du Pasquier et al. 2006; Nakajima and Yaoita 2003).

We assessed T_3_-dependent tail shrinkage by measuring the area of the tail fin over a 4-day period, which is generally the time it takes for the fin to completely resorb in our assay. Fin resorption is the first step in visible tail metamorphosis, is amenable to quantitation by morphometric analysis, and is representative of the overall tail shrinkage process. The *ex vivo* tail culture allows for the very controlled and T_3_-driven metamorphosis of an entire tissue and, because the tadpole skin is so permeable, both T_3_ and As are able to diffuse into the tissue from the media (Lim et al. 2002; Veldhoen and Helbing 2001). Tail resorption is completely dependent on T_3_ acting as a ligand for TR and trans-activating genes necessary for resorption, as shown in previous studies including those using an antagonist to TR, which blocks these processes (Lim et al. 2002). The lowest As concentration that had an effect on tail fin resorption (0.1 μM) did not follow the dose-dependent pattern of all the higher As concentrations. (Figure 8). This biphasic pattern was highly repeatable and statistically significant, indicating two different dose responses over this range and suggesting perhaps two different mechanisms of action underlying these effects. Indeed, two different mechanisms of interference could lead to the same overt results (i.e., less fin shrinkage).

Tail resorption is only one piece of the complete transformation of the aquatic tadpole into the terrestrial frog (Das et al. 2006; Dodd and Dodd 1976). Tissues in the animal are completely remodeled to function on land. In the space of a few weeks, body parts are generated (limbs) while others completely resorb (tail); cell death, growth, and differentiation can occur simultaneously in a single tissue (intestine, eye, blood, skin). All this transformation is directed by TH. If TH is removed, metamorphosis does not happen and a continuously growing tadpole will result (Allen 1916; Dodd and Dodd 1976). When TR is expressed and T_3_ is produced, the program for the transcription of genes necessary for metamorphosis in any tissue is ready to proceed. This model has relevance to human development because the plasma levels of TH spike in humans during the perinatal period (6 months of gestation through several months postnatal), which correlates temporally with the TH spike in amphibians during metamorphosis (Shi et al. 2002). Extreme thyroid deficiency in humans at birth leads to cretinism, with characteristic mental retardation, short stature, and hearing loss. These severe deficiencies are detected at birth and can be quickly treated with exogenous hormone, but subtler effects may not be as evident at birth (Galton 2005; Oppenheimer and Samuels 1983; Raz and Kelley 1997). Indeed, subtle effects on cognitive function have been noted in epidemiology studies of children exposed to excess As in drinking water (Wasserman et al. 2004). Studies showing disruption of thyroid hormone functions after As exposure in rodents include an increase in thyroid cancer (Yamamoto 1995), a synergistic effect between As and TH on oxidative stress (Allen and Rana 2003), and a disregulation of deiodinase levels in fetal brain with concurrent As exposure and selenium depletion (Miyazaki et al. 2005).

In the present study we have demonstrated that As can act as a potent endocrine disruptor not only for the entire SR family but also for two important members of the larger nuclear hormone receptor superfamily. It seems likely, based on the ubiquity of these effects and their likely common or shared mechanism(s), that As will also have similar effects on other members of this large hormone receptor superfamily. It will be important to determine the extent to which this occurs and under what conditions, but also to investigate the biological consequences of such effects. Including the present study, we have demonstrated in two different *in vivo* systems that there are specific and predictable pathophysiologic effects of endocrine disruption by As. We previously reported that As has profound effects on the GR-dependent freshwater-to-saltwater transition of killifish (*Fundulus heteroclitus*) (Shaw et al. 2007; Stanton et al. 2006).

Exposure to excess As in drinking water has been associated with an extensive and growing list of disease risks. Given the important and myriad roles of hormones and their receptors in normal physiology and in the pathophysiology of these and other diseases, it is likely that endocrine disruption by As plays an important role. Previously, it was thought that because As does not significantly accumulate in the body, unlike persistent organics or metals such as mercury and lead, and also does not cause DNA damage or mutations, its effects on disease risk might also be transient and reversible. However, such effects of As on hormone signaling at key developmental or differentiation points might be expected to result in long-term effects related to epi-genetic phenomena such as imprinting. In this regard, it is interesting to note several recent studies that support this idea (Liu et al. 2006a; Shen et al. 2006; Waalkes et al. 2004a). Among other interesting changes, a profound up-regulation of ER expression has been reported in livers of adult mice exposed *in utero* to As, in addition to their increased incidence of liver tumors (Chen et al. 2004; Liu et al. 2006b; Waalkes et al. 2004b). Two recent epidemiology studies of humans exposed to As in drinking water were also provocative (Smith et al. 2006; Wasserman et al. 2004). These studies suggest that there can be significant long-term consequences of exposing young or developing individuals to As during critical periods of development. We propose that disruption of hormone signaling is likely to be an important component of these effects. Thus, understanding the role of As as an endocrine disruptor will be important for assessing the overall impact of As on human health and in developing appropriate risk assessment paradigms that are protective of human health.
